# Initial Development of a Patient Reported Experience Measure for Older Adults Attending the Emergency Department: Part I—Interviews with Service Users

**DOI:** 10.3390/healthcare11050717

**Published:** 2023-02-28

**Authors:** Blair Graham, Jason E. Smith, Pam Nelmes, Rosalyn Squire, Jos M. Latour

**Affiliations:** 1 School of Nursing and Midwifery, Faculty of Health, University of Plymouth, Plymouth PL4 8AA, UK; 2 Department of Emergency Medicine, University Hospitals Plymouth NHS Trust, Plymouth PL6 8DH, UK; 3 School of Nursing, Midwifery and Paramedicine, Faculty of Health Sciences, Curtin University, Perth 6102, Australia

**Keywords:** emergency medicine, emergency department, aged, patient experience, communication

## Abstract

Older adults are a major Emergency Department (ED) user group who may be especially vulnerable to the consequences of crowding and sub-optimal care. Patient experience is a critical component of high-quality ED care and has previously been conceptualised using a framework focusing on patients’ needs. This study aimed to explore the experiences of older adults attending the ED in relation to the existing needs-based framework. Semi-structured interviews were conducted during an emergency care episode with 24 participants aged over 65 years in a United Kingdom ED with an annual census ~100,000. Questions exploring patient experiences of care confirmed that meeting the communication, care, waiting, physical, and environmental needs were prominent determinants of experience for older adults. A further analytical theme emerged which did not align to the existing framework, focused on ‘team attitudes and values’. This study builds on existing knowledge relating to the experience of older adults in the ED. In addition, data will also contribute to the generation of candidate items for the development of a patient reported experience measure for older adults attending the ED.

## 1. Introduction

Older adults aged over 65 years are a major user group of Emergency Departments (EDs), comprising over 29% of attendances in a recent UK retrospective cohort study [1]. In many countries, the number of older adults attending the ED is increasing above predictions based on population size alone. Contributors may include increasing comorbidity, gaps in primary healthcare, and increasing numbers of repeated ED attendances among frail older adults [2,3]. Older adults are more likely to present to the ED with high acuity conditions [4], yet atypical presentations and non-specific symptomatology are also more common. This clinical complexity may contribute to increased healthcare costs and ED resource utilisation [5]. Furthermore, older adults more frequently suffer from background comorbidities, long-term conditions, and have more nursing care requirements [6,7]. For all of these reasons, older adults encounter above-average ED length-of-stay (LOS) [8] and are at increased odds of requiring hospital admission [9].

In addition to the range of challenges posed by older adults, wider demand for emergency care is being encountered internationally [10]. Many systems have failed to keep pace with this demand. As a result, ED crowding is now a significant public health concern, responsible for an estimated 4000 excess deaths in the UK alone during 2020–2021 [11]. Older adults fare unfavourably when treated within a pressured emergency care system and are significantly more likely than the general population to suffer 30-day mortality following a protracted ED LOS. The reasons for this are likely to be complex, but delayed medication administration, poor continuity of care, increased risk of nosocomial infections, and circadian disruption resulting from sleep deprivation have all been postulated [12]. The proportion of over 65s is projected to double in most nations before 2050 [13]. Consequently, the demand on ED services from older adults is likely to reflect this trend. To meet the needs of older adults as a predominant ED user group and reduce the risks that they may encounter when accessing care, there is a pressing requirement to ensure that the ED environment and care processes are thoroughly considered and fit for purpose.

Effective and meaningful measurement of the quality of emergency care is essential to enable comparison between different settings and drive improvements in clinical outcomes, patient experience, and safety. In many healthcare systems, this continues to be achieved using process-centred performance metrics. For example, recently introduced ED performance standards in England include timely ambulance handover, time-to-triage assessment, and ED LOS [14]. Whereas some performance metrics—such as time-based targets—have been demonstrated to lead to meaningful improvements in some aspects of care [15], a limitation is that they may fail to effectively capture outcomes of care that matter most to patients. To this end, the International Federation for Emergency Medicine (IFEM) recommends the adoption of both Patient Reported Outcome Measures (PROMs) and Patient Reported Experience Measures (PREMs) within their recently updated framework on quality and safety in emergency medicine [16]. Patient experience is recognised within prominent definitions of quality of care and is associated with improved clinical outcomes and patient safety [17,18]. However, meaningful evaluation of patient experience can be challenging. For example, surveys are frequently undermined by poor response rate and issues with face validity and reliability [19,20]. Furthermore, generic surveys may not identify specific vulnerabilities in care processes from the patients’ perspective, representing a missed opportunity to effect change and improve services [21].

Patient reported experience measures (PREMs) are psychometrically validated questionnaires that are directly reported by patients and aim to provide standardised evaluation of individual experiences of care [22]. To ensure content validity, PREMs should be developed in conjunction with patients and care providers to capture aspects of care that are important [23]. Several PREMs relating to ED care have been developed, although limitations of these instruments include uncertain validity, reliability, and responsiveness [20]. No instrument has yet been developed to specifically measure the experience of older adults, aged 65 years and above, in the ED.

The overall aim of our project is to develop and validate a PREM to address the unmet need of an instrument for older adults in the ED, known as the Patient Reported Experience Measure for patients attending the Emergency Department aged over 65 (PREM-ED 65). From the outset, the development process for PREM-ED 65 is designed to ensure the final instrument meets the internationally accepted COnsensus-based Standards for the selection of health Measurement INstruments (COSMIN) guidelines [24]. PREM development is planned using a step-wise mixed methods approach. This is summarised in Table 1.

In order to derive suitable candidate questionnaire items, an initial systematic review of patient experiences in the ED has been conducted. This has resulted in a conceptual framework to guide understanding of patient experience in the ED. This framework is based around the needs of ED patients and includes five analytical themes: communication needs, emotional needs, care needs, waiting needs, and physical/environmental needs [25]. The next step of the PREM-ED 65 development process is to consider how the five analytical themes are experienced by older adults attending the ED and health professionals responsible for delivering emergency care.

We conducted a two-part qualitative study aiming to explore the experiences of older adults attending the ED (Part I), and the experiences of emergency department healthcare professionals (see Part II—Focus Groups with Professional Caregivers). Part I aims to explore older adults’ experiences of an ED visit in relation to the pre-established conceptual framework and to determine if any additional analytical themes emerge.

## 2. Materials and Methods

We adopted a qualitative design using semi-structured interviews conducted with older adults aged ≥ 65 years during their ED visit. This study is reported following the COnsolidated criteria for REporting Qualitative research (COREQ) checklist [26]. Ethical approval was prospectively obtained from the UK Health Research Authority (18/LO/1194) and institutional approval from the University of Plymouth (17/18-973).

### 2.1. Research Team and Reflexivity

Interviews were conducted by a male-identifying researcher (BG) who is an academic emergency physician and research fellow with prior experience and formal training in qualitative research methods. Two female-identifying clinical academic nurses (PN, RS) assisted in the transcription and initial coding of data. A male-identifying professor in emergency medicine (JES) and male-identifying clinical nursing professor (JML) with extensive experience of quantitative and qualitative research in acute care settings were involved in data analysis.

Principles of rigour and trustworthiness for qualitative research were applied [27]. Researchers considered their own clinical experiences and the need to exclude these during the analysis and interpretation of findings. The first author maintained reflexive notes and discussed perceptions with co-researchers during the study. No relationship was established with participants prior to study commencement. Standard information was issued to all participants prior to recruitment and consent. Participants were told the research’s purpose was to inform PREM development as part of the lead researcher’s PhD study.

### 2.2. Theoretical Framework

The interviews were deductive, informed by the overarching definition of quality of care proposed by Darzi [17]. This definition encompasses three domains: patient experience, clinical effectiveness, and patient safety. Although our interviews focus on capturing the experiences of people in ED, the use of these three domains also allows for the exploration of clinical expectations and perceptions of safety in the ED, which are known to be related to experience. The three domains of the quality-of-care definition were used to formulate the interview questions in the interview guide.

### 2.3. Participant Selection

Inclusion criteria were adults aged ≥ 65 years attending the ED. Patients who lacked mental capacity to give informed consent [28], who were too unwell to participate or required immediate life-saving treatment (‘*Category 1*’ triage category), did not speak English, or were in police/prison custody were excluded from the study.

A purposive sampling strategy was used to encourage recruitment of a representative cross-section of patients attending the ED. Patients were sampled based on the presence of either traumatic injury or medical illness, age group, clinical frailty score [29] and acuity (Table 2). A sampling matrix was used to support the inclusion of patients from each sampling category.

Twenty-four patients were recruited during daytime hours (0800–1800) between September 2018 and April 2019. In addition, a single patient was recruited but then withdrawn prior to the interview occurring, due to them being transferred away from the ED. The computerised ED administration system was used to screen potentially eligible patients. Once the patient’s attending clinician indicated that treatment was complete, the clinician was approached and asked to give their assent for the patient to be invited to participate. Eligible patients were then approached by the lead researcher who presented them with verbal information about the study, and a written patient information sheet.

Sampling was conducted until the researchers were satisfied that sufficient data had been collected through the interview process to reach ‘data saturation’. This suggests the collection of further data is unlikely to add value [30]. Based on insights from the literature, it was estimated that between 20 and 30 participants would be needed [31].

Patients who were approached but did not wish to participate were not recorded and not recruited into the study.

### 2.4. Setting

Interviews were held within a single ED in the South West of England (Annual Census ~100,000/annum). As a regional major trauma centre, the ED receives patients from urban and rural settings within a wide geographical catchment (population ~1.65 million), and notably has a higher-than-average proportion of attendances from older adults. ‘In situ’ interviews, conducted within the ED during an acute care episode, were selected as the preferred approach to maximise ecological validity whilst minimising recall bias. A range of clinical areas within the ED were utilised for interviews and included bedspaces, ambulatory spaces, relatives’ rooms, and the attached short-stay clinical decision unit. Where a patient was identified for interview but was transferred to an inpatient setting before an interview within the ED was possible, the study protocol allowed for interviews to take place on the receiving inpatient ward, provided this was clinically appropriate and within 24 h of admission.

### 2.5. Data Collection

The interviewer used an interview guide (Table 3). Three questions were posed to all participants, each exploring one of the three domains of Darzi’s original definition of quality of care [17]. Prompts were prospectively developed from our understanding of the existing literature and were suggested as part of the question guide, although the interviewer could deviate beyond these if discussion deemed it necessary.

Interviews were audio recorded using a digital voice recorder with noise cancelling technology and a dual lapel microphone to ensure clarity. Interviews were transcribed verbatim, and a proportion of transcripts were cross-checked to ensure accuracy. Additional field notes were taken to capture appropriate non-verbal, paralinguistic communication, where appropriate.

### 2.6. Data Analysis

Transcripts were uploaded into NVivo Version 12 (QSR International, Massachusetts, 2012), a software programme used for qualitative analysis. Framework analysis following a mixed inductive-deductive approach was adopted, following the seven steps described by Gale et al. [32]. The first two steps of this approach are *transcription* and *familiarisation.* For this, two members of the research team (BG, RS) took responsibility for transcription, and worked collaboratively to cross-check each other’s work. This ensured both accuracy of transcription and familiarity with the interview content. The third step is *coding*. Interview transcripts were selected and open coded using an inductive approach. A modified approach to step four—*developing an analytical framework*—was adopted. Rather than develop a new framework, we adopted our pre-existing needs-based framework [25]. As such, for step five—*applying the analytical framework*—we switched to a deductive approach to index codes under the exisitng analytical themes based on ‘best fit’. For the final two steps—*charting* and *interpreting data*—all researchers met to review data, summarise findings and identify illustrative quotations.

Where data was deemed not compatible with existing themes, these were discussed between the researchers and either a pre-existing theme was agreed, or a new theme was formulated and agreed.

### 2.7. Presentation of Findings

Presentation of findings includes description of the study participants; coding and emerging themes, including the frequency (prevalence) of statements aligned to each theme; and detailed discussion of findings by analytical theme, supported by illustrative quotations.

## 3. Findings

### 3.1. Description of the Study Participants

Twenty-four participants were recruited and completed the interviews. Mean age was 74 years (range 65–91 years). A larger proportion of participants were female (62.5% versus 37.5% male). Almost all patients declared at least one long-term condition or co-morbidity (95.8%). Patients had a range of education levels ranging from no formal qualifications to professional qualifications. Participants were recruited from across the acuity spectrum and included a majority of ‘very urgent’ (Category 2) and ‘urgent’ (Category 3) presentations (35% and 54%, respectively). Two-thirds of patients (66.6%) presented with a non-traumatic medical complaint. Most patients had lower or moderate levels of frailty as assessed by the clinical frailty score (mean CFS = 2.6 out of 9, range 1–6). All but a single patient lived in his or her own accommodation (Table 4). An overview of individual participant characteristics can be found in Electronic Supplementary Material (Table S1).

### 3.2. Coding and Emerging Themes

Framework analysis of transcripts was conducted, and statements assigned to an existing analytical theme where appropriate. Five hundred statements were identified which were directly aligned to experience. Of these, 452 statements were organised under one of the five existing analytical themes within the established conceptual framework. These were most prevalent around waiting needs (146 statements), followed by statements related to ‘communication needs’ (125 statements). ‘Emotional’ and ‘physical/environmental’ needs were evenly distributed (67 and 66 statements, respectively). Statements related to ‘care needs’ were slightly less prevalent (48 statements).

During the framework analysis an additional analytical theme emerged, relating to *Attitudes and Values of the Team*. This was initially identified within the interviews data, to accommodate 59 unique statements relating to patients’ perceptions of the ED team members, teamwork, and professionalism.

The data identified several new sub-themes were also identified. These are presented in Table 5 and include Social Communication (under communication needs), Reassurance (under emotional needs), and Waiting Experiences (under waiting needs).

Selected statements within this report are presented with reference to the study participant number (Pn).

### 3.3. Presentation of Findings

#### 3.3.1. Communication Needs

The theme of Communication Needs encompasses statements that relate to patient-provider communication. These are divided into inter-personal communication, which consists of components of experience typically featured within a healthcare consultation, informational communication, which consists of the giving and receiving of information (for example, discharge instructions), and the new subtheme social communication, which consists of components of communication such as conversation not formally considered within consultation frameworks or models.

##### Interpersonal Communication

The interviews confirmed that patients placed immense value on inter-personal communication with care providers and the wider ED team. Patients preferred for staff to display a communication style that was calm, unrushed, and polite:

*Well…..they spoke to you in a good way…. they sounded as if they were interested and they weren’t rushing… they seemed genuinely polite*

(P13)

Patients were able to detect when staff communicated with them in a way that encouraged them to feel valued as individuals, and this positively affected their perceptions of care:

*They made you feel that you were an individual kind of thing. They made you feel….you know, that you were on a level with any any dignitary paying a lot of money*

(P18)

Most patients confirmed that staff introduced themselves by name and role, and this was viewed as beneficial and could help demystify roles, especially in instances where the patient was unfamiliar with the ED environment or had not attended before. However, a succession of staff introductions could be confusing where patients were presented with multiple new members of staff in a short space of time, as was reported by one patient:

*I find it very confusing I mean there were three or four nurses that came in and told me their names and then the consultant came in and said good morning my name is blur blur and before he started on me he disappeared and I haven’t seen him again!*

(P4)

Similarly, patients also wanted to know the role of staff members, particularly when they were administering a task. Patients understood that staff had a job to do including tasks that would potentially be unpleasant or uncomfortable. A friendly attitude meant that patients readily accepted such experiences, which might otherwise be aversive. Patients were sometimes cognisant of conversations happening around them in the clinical environment. In such instances, they wanted to be active participants in discussions relating to their care rather than passive listeners. Being talked about without the opportunity to contribute to discussions during a clinical handover was perceived as undermining by one participant:

*I’m not stupid… they’re talking about bed ‘G4’…I’m G4! I’m not stupid, it’s not rocket science. Weird. Just weird… I’d like to be in on the conversation, rather than just hearing in the distance.*

(P17)

Some of the older participants reported that communication with medical staff had evolved compared to their historical experiences. Demonstrating politeness and offering explanations as part of the ED consultation positively affected patients’ experiences:

*cos no matter what you ask them, they’re there they speak to you politely and they don’t dismiss you. The doctors explain everything to you which they never did years ago. So you know exactly what’s going on and you know exactly what’s wrong with you.*

(P24)

*In the olden days if you came into hospital everything was kept secret (laughs) if you know what I mean. But today it’s quite relevant to let people know what’s going to happen and what’s going on.*

(P05)

Sensory problems and impairments are frequent amongst the older adult population. Recognition of a hearing impairment and adaptation of communication strategy was essential in facilitating a positive experience for one participant but prevented communication of a test result for another. Regarding providing information, patients valued understandable and appropriately detailed explanations. Repetition of questioning was also noted as a common feature of the ED consult, which could adversely affect experience. When explanations were provided which were too advanced for a patient to fully understand, particularly where this occurred without checking understanding, this could result in some frustration as expressed by one participant:

*Information needs to be broken down to suit your average patient. You know, we’re not all nurses or doctors. And I’ve had this for years, people have come along and said this and that and I’ve not understood it. It doesn’t work all the time for me.*

(P20)

Although, reflecting the local population, participants were predominantly White British, the importance of cultural and language recognition was noted by one patient who had a Welsh background:

*I got on with one particular nurse cos she was Welsh … she spoke in welsh*

(P14)

##### Informational Communication

In addition to valuing interpersonal aspects of communication, patients had a great desire to be kept informed of their situation and the progress of their ED journey. Although verbal informational communication was considered most frequently by participants, other methods of delivery including written information and multi-media delivery—for example, using ‘on screen’ presentations in the waiting area was also desirable. Positive experiences of informational communication also included the progress of tests and investigations, and communication of ‘next steps’ during physical procedures:

*The doctor came in this morning and told me about my blood test … I’ve got to go down for an X-ray and I’ve got to go for scans.*

(P05)

##### Social Communication

Although not routinely considered as a part of consultation or communication skills frameworks, social communication was mentioned by some patients as important to their experience and is therefore considered within this synthesis. Patients expected staff to be friendly towards them and use positive body language such as smiling:

*It’s nice to have a smiling face and just to be sociable and polite and I hope I’m the same to them.*

(P07)

The use of humour by staff was seen as very welcome by some patients. Despite this, one patient who was previously a senior nurse remarked that the use of humour should adhere to professional boundaries and that using colloquial terms of endearment, rather than addressing patients by their name, was inappropriate:

*Not being too familiar, you know … [I don’t like] being called ‘babe’.*

(P02)

#### 3.3.2. Emotional Needs

In the original framework, the theme Emotional Needs was divided into sub-themes encompassing coping with uncertainty, recognition of suffering and empowerment. Following framework analysis, an additional sub-theme of reassurance was added.

##### Coping with Uncertainty

Perceptions of uncertainty were mentioned by several study participants and could be a fundamental emotion associated with attending the ED, particularly if not familiar with the setting previously. Uncertainty also elicited feelings of vulnerability when patients were unsure of outstanding investigation or treatment plans:

*“I was told ‘do this and take your dressing gown off and put a nightdress on and we will do an ECG’ or something like that, and I thought what’s an ECG, you know?… it didn’t worry me, but I was concerned what they were going to do!”*

(P06)

Conversely, it was recognised that perceptions of uncertainty could be effectively managed by simple interventions such as keeping patients informed of the next stages of care. Even where patients were accepting of uncertainty, they still desired basic information, such as whether they would be staying overnight:

*I did ask someone if I was stopping overnight, cos nobody had said, perhaps they didn’t know but you know, sometimes it’s helpful.*

(P13)

##### Recognition of Suffering

Recognition of suffering was addressed by several patients. This extends beyond pain, to include recognition and attention to other forms of suffering and distress. One patient, who felt her suffering was not recognised in ED, was able to give an example of an experience in oncology:

*… with Oncology, immediately when you went in there was a member of staff with you. Whether it was a ward assistant of whether it was a nurse, somebody was with you, talking through your problems, how you felt and so you felt hum, you felt loved and comforted whereas I don’t [in ED]*

(P11)

##### Empowerment

Empowerment is defined by the European Patient Forum as “any process that helps people gain control over their own lives, and increases their capacity to act on issues that they themselves define as important” [33]. Staff provided empowerment to patients by making them feel like individuals, legitimising their reasons for attendance:

*Yes I feel like my concerns have been taken seriously, yes I do.*

(P12)

Although some older adults were appreciative of having appropriate say in their care decisions, others recognised the potential importance of following clinical advice, for example, surrounding a decision to admit to hospital:

*It’s no good talking to medical staff and completely ignoring what they have to tell you, and if they advise that I should be over night, because they want to find out why I’ve gone down twice … it’s an obvious answer isn’t it.*

(P19)

On occasion the environment of the ED could be physically disempowering. One patient reflected humorously on her experience of being attached to monitoring equipment, likening this to ‘being chained up like Houdini’ (INT_14). Some patients expressed fear of judgement from staff, which could be disempowering and affect their ability to make decisions:

*You can see them thinking ‘how the fuck did he manage to do that?!’ (laughs) … do you know what I mean? And you feel as though you’re being judged as a village idiot.*

(P17)

##### Provision of Reassurance

During analysis, several statements relating to the provision of reassurance were encountered. Many patients viewed the provision of reassurance as a key positive determinant of their experience, and a sense of reassurance was often conveyed through good patient-provider communication:

*[The staff are] quite happy, they introduce themselves, they sit down … they talk to you as a human being. That they reassure you. That’s quite nice.*

(P04)

Reassurance could also be provided as an active process; for example, by the positive actions of staff, showing thoroughness and diligence, or in one case, giving passive reassurance that material property was safe:

#### 3.3.3. Care Needs

The third analytical theme, Care Needs is sub-divided into symptom relief and procedural care.

##### Symptom Relief

With regards to symptom relief, pain management was central to achieving a good experience. Patients made clear that they expected pain assessment and the provision of analgesia early on in their ED stay as a priority:

*Keeping the pain at bay, really, is the big thing*

(P16)

Staff met patients care needs by maintaining comfort during potentially painful procedures, for example IV cannulation. Explanation of procedures was reported to be extremely helpful, including effects on physical dignity:

*I had to take my bra off you see, and when [the nurse] was putting the pads on I said I’m sorry…. He said ‘don’t worry about that—I won’t be worried about that!’ (chuckle) He was very kind about that … I think when you get a bit older you get a bit embarrassed*.

(P03)

##### Procedural Care

Procedural care is defined as care delivered during medical or nursing procedures. Competence was valued highly in relation to this sub-theme. Patients were perceptive of when they were being seen by a junior member of staff and whilst they were happy to be attended by trainees, desired to have the attention of more senior clinicians. One patient, who attended following a therapeutic excess of paracetamol was a critical observer of the doctor who made an antidote drug calculation. Even so, the friendly nature of the encounter mitigated any negative effect on overall experience:

*Well I think he was struggling to calculate whether the amount of paracetamol taken was too much, I think he really struggled with the calculation*

*(Interviewer: Did That affect your experience?)*

*No, not really because he was so nice. He came to me later on and apologised and said ‘ I’m so sorry for what you’ve gone through today’ and that was so nice… yeah, so that was OK.*

(P22)

#### 3.3.4. Waiting Needs

The theme Waiting Needs was subcategorised into the comfort (associated with waiting), impact of crowding and a new subtheme, waiting experience:

##### Comfort Whilst Waiting

Waiting could be uncomfortable and witnessing other patients’ suffering distressing. However, these negative aspects of the experience could be mitigated through accepting shared experiences, resulting in camaraderie amongst patients:

*“I think there was a bit of … you know … the patients forming a group, and the doctors and nursing staff forming a group. We were all in the same boat.”*

(P22)

There was an awareness of the breadth of acuity presenting to the ED (“….you’re dealing with the serious to ridiculous, aren’t you?! (P11), that patients underwent triage, and that having a lower triage category could necessitate a longer wait. However, patients wanted to have accurate information about waiting times, which were not always provided:

*“… they were giving answers they thought we wanted to hear, for example, ‘ somebody is coming’… well… somebody wasn’t coming, and that was an issue! Somebody was coming 2 h later!(laughs) … I think I’d rather be told the real state of things.”*

(P11)

##### Impact of Crowding

The waiting room could be cramped, and being near other unwell patients was intimidating and upsetting for some. Patients frequently reported physical discomfort whilst waiting, which was due to the metal chairs and gurneys in the department:

*The seats… oh the seats were dreadful! Someone was lying on the floor in preference to sitting on the chairs because they were in such a lot of pain!*

(P22)

Providing basic comfort measures was a positive determinant of experience; even providing blankets improved the experience for some patients. Conversely, ambient temperature was problematic for some patients, who reported thirst and headaches because of heat and inability to access refreshments:

*I was cold…and I had a blanket brought to me straight away. I thought it was lovely.*

(P04)

*It would have been nice to have water. It’s warm in here. I’ve got a bit of a headache now and I think it’s just the heat.*

(P21)

Being cared for in corridor spaces could provoke significant anxiety for patients, who perceived this experience as unsafe and undignified:

*Just the thought of having to wait in the corridor … just waiting there. No, I didn’t like that. Because everybody’s walking by you and they’re looking at you as if to say ‘what’s wrong with her’?!*

(P04)

##### Waiting Experience

Study participants recognised the necessity of waiting for their care to be initiated and were understanding and accommodating of the need to wait. Even so, negative consequences of waiting were reported and extended beyond boredom and frustration. On occasion, participants found the waiting experience to be intimidating, particularly when in close confines with other patients and those who were acutely unwell. Patients expected waiting to feature as a part of their ED experience, and were tolerant of the need to wait, even where this was prolonged:

*Waiting is part of life’s rich tapestry isn’t it? …. You put up with it.*

(P20)

#### 3.3.5. Physical and Environmental Needs

The theme Physical and Environmental Needs describes how the physical environment of the ED influences patient experience. This includes the provision of fundamental needs such as refreshments. Interaction with the environment formed an important determinant experience for many patients and included the subtheme comfort (associated with physical needs).

The presence of clear signage to the department and reception was noted to be absent, which was a negative determinant of experience. Cleanliness and hygiene of the ED environment was a crucial factor in the experience of patients:

*[The environment] has got to be clean, otherwise you get all of the bugs, don’t you?*

(P03)

There were several aspects of the ED physical environment which could be negative determinants of experience. For example, one patient remarked that the environment of the waiting area—including the presence of others who were intoxicated, violent or agitated—could be unsettling and frightening:

*No. Just that … people screaming … you hear it in here … men, screaming. Alcoholics who want a drink. And that’s upsetting when you’re trying to go to sleep. And you don’t feel safe then because you think are they going to come around here, you know.*

(P04)

Background noise could be problematic, particularly for patients with pre-existing hearing difficulties. Monitor alarms are a constant presence in some parts of the ED, and study participants experienced this as noise pollution.

*“…. all the buzzers and beeps… it is like having a train at the bottom of your garden.”*

(P23)

##### Comfort (Associated with Physical Needs)

In terms of basic needs provision, patients appreciated the provision of refreshments and noticed when these were not offered:

*“When we came in [to ED] we were offered tea, and it went so much more quickly”*

(P01)

#### 3.3.6. Attitudes and Values of the Team

As many statements related to perceptions of teamwork and staff attitudes and professionalism, a new analytical theme, labelled ‘Attitudes and Values of the Team’, and was added to the existing framework. The two subthemes associated with this theme are perceptions of teamwork and staff behaviours.

##### Perceptions of Teamwork

Patients were active observers of team-based processes, and took reassurance from witnessing effective communication between different team members:

*Yeah … when you’re on a trolley you tend to watch. And what I noticed was how they were talking and passing information. And I thought that was brilliant. They knew exactly where to go, you could see it.*

(P04)

##### Staff Attitudes and Professionalism

Patients expected professionalism from the wider team, and had a sense of the staff members working together for them as an individual patient:

*I felt that everyone was part of a team that had one aim in focus which was to look after the patient which was me.*

(P17)

Values that patients perceived staff exhibited during care episodes that contributed to a positive experience included kindness, politeness and an approachability were reported:

*“Everybody’s kindness and professionalism stood out today.*

*(Interviewer: And what is it that gives you that impression of kindness?)*

*… well, it’s staff being attentive and … the fact that I’ve asked questions and the staff have*

*always answered politely without being harassed. I really feel like I can approach them.”*

(P09)

However, some patients did find the number of team members and different roles confusing:

*There is so many people doing so many different jobs, each with their own coloured uniform, and you just wonder what they were doing.*

(P22)

Regarding continuity of care, some patients were aware of team shift changeovers and felt a burden when establishing a rapport with a new team. Shift changeovers during a phase of care could increase their sense of patients’ vulnerability:

*“…knowing that when staff finish their shift … that they are going…that they are passing you over, you lose that continuity. I know they all work as a team… but [as a patient] you may have to re-establish something emotionally [with the new team]”*

(P09)

## 4. Discussion

This study aimed to understand the experiences of older adults in the ED. Framework analysis using a combined inductive-deductive approach [32] confirms the conceptual validity of a ‘needs based’ framework amongst older adults in a UK ED setting. Statements were notably prevalent around waiting needs and ‘communication needs’. There was also sufficient quality and quantity of statements to confirm the presence of the three remaining analytical themes. It should be noted that reporting of prevalence of themes within qualitative research is controversial and that the number of statements related to a theme is not necessarily proportional to its significance [34,35]. However, in our case, presenting frequencies of statements related to the themes provides important assurance that data has been explored in its entirety. Accordingly, this ensures data used to inform item generation for PREM-ED 65 accurately reflects the full breadth of patient experiences reported in the interviews [36].

In addition to the existing five analytical themes, a new descriptive theme emerged, describing the role of staff professionalism and teamwork in contributing to the patient experience. This is supported by the previous literature suggesting that patients are direct observers of team-based processes [37,38] and that observation of constructive teamwork is a positive determinant of patient experience in the ED.

Patients in our study have provided narratives that will contribute to the further development of PREM-ED 65. This study builds on the body of the existing literature emphasising patients’ desire to have their basic human needs and comfort addressed during emergency care episodes [25,39,40]. If aiming to optimise patient experience, the provision of humanistic, holistic care should be considered an important caring aspect of the ED alongside the clinical objectives. Specifically, in our study, participants also expected their dignity and privacy to be respected wherever possible. Including these factors in a PREM is desirable from the evidence of our patient interviews and findings.

Facilitators of a positive patient experience that were identified included personable communication, the provision of timely information relating to the progress of clinical assessment/onward disposition, and measures to promote both physical and emotional comfort. Although it is useful to conceptualise these facilitators as discrete elements of patient experience—for example, when identifying a focus for service improvement—they may not be mutually exclusive and can overlap. For example, when looking at facilitators related to the analytical theme of *social communication*, providers’ use of humour may extend beyond a purely social function to promoting the development of trustful patient–provider relationships [41,42]. Hence, in this case, social communication may have a role in meeting both *communication* and *emotional* needs. The potential for themes to interact and overlap should be considered when applying the original needs-based framework to the real-world setting.

Although the focus of participants’ discussion focused on their perceptions of relational aspects of care, as opposed to technical care elements, the need for prompt pain relief and symptom control was a common topic within our study participants. This is also recognised in the literature, for example in a prospective observational study of pain management in the ED by Van Zanden and colleagues [43] where 43.7% of patients arriving in the ED desired pain relief, and the provision of pain relief was associated with higher satisfaction. Pain was also highlighted in a recent qualitative study exploring patients’ experiences in an Australian ED [40]. Many patients in this study described pain as a memorable aspect of their ED visit. In contrast to the positive experience, patients who do not receive timely pain medication had negative ED experiences, as confirmed elsewhere in the literature.

Our interviews suggest that both the length of wait and care delivered whilst waiting for medical assessment and treatment form an important determinant of older adults’ experience in the ED. Indeed, waiting is ubiquitously associated with accessing emergency care, and the literature suggests that patients often expect a long ED waiting time [40]. In the UK setting, older patients wait longer, and have a prolonged stay in the ED compared to younger patients [1]. However, there may be some international variation in wait times experienced by older adults [44,45]. As such, it is important that a PREM aimed at older adults examines the experience of waiting, which may give locally relevant insights into where improvement would be beneficial.

### Strengths and Limitations

This study represents a unique attempt to interview patients ‘in situ’, within the ED, during their stay. Ecological validity is a concept originally described in the social sciences following recognition that experimental conditions must mimic the ‘real world’ to promote external validity [46]. Importantly, ecological validity may also be impaired if interviews and surveys are conducted away from the setting of interest [47]. To this end, in situ interviews maximise ecological validity whilst also minimising recall bias, as patients are reporting experiences from ‘in the moment’, as they are lived. The effect of recall bias may be especially significant where the time spent in the ED itself is short; in this situation, self-reported perceptions of a care episode are likely to be affected by subsequent admission to other hospital departments. The available literature also suggests that recall bias may be more pronounced in older adults, who have been noted to recall events more positively in hindsight [48]. Conversely, potential limitations of the ‘in situ’ approach may include concerns about privacy, confidentiality, and the effect of disclosing information on care. To mitigate against these potential effects, the informed consent procedure included explicitly informing participants that information would not be shared with caregivers. Furthermore, interviews were conducted in private settings within the ED, wherever possible. Another limitation of our study is that it did not include older patients meeting the Rockwood criteria of ‘very frail’. However, a recent qualitative study was conducted across three EDs in the UK and specifically recruiting older adults with frailty (Rockwood CFS > 5). The findings of this study derived some similar themes, including information and communication in the ED, time waiting in the ED, and environment/personal comfort [49].

The general experience of the researchers towards the ‘in situ’ approach to interviewing patients in the ED is a positive one. However, those utilising this approach in the future may consider pre-arranging a private space, away from the immediate clinical area, in which to conduct the actual interviews. This may optimise interviewer-interviewee communication and enhance comfort for both parties. Strategies to promote representativeness of the sample, including recruitment of ‘hard to reach’ groups, should also be considered. In our experience, recruitment of very frail older adults was difficult. This is also reported in the literature, which suggests recruitment of this group may be improved by considering when to approach very frail participants (e.g., following a period of rest), building personal rapport when explaining the project, giving them more time to consider participation, and—with their agreement—discussing with, or indeed involving, relatives and trusted friends [50]. Although the time required to implement these strategies may seem at odds with ‘in situ’ interviews conducted during an ED stay, many older adults currently experience protracted waits for admission, increasing the potential relevance and feasibility in the current context [51].

Finally, it should be noted that patient interviews were conducted prior to, and were therefore not influenced by, the COVID-19 pandemic.

In summary, these findings build on our previous conceptual framework. This confirms face validity amongst an older adult population attending the ED. In qualitative research, triangulation refers to the use of multiple methods to develop a more comprehensive understanding of phenomena [52]. Data from this study will be triangulated with both the existing literature [25] and an accompanying focus groups study with healthcare professionals. (Part II) This will yield a comprehensive list of candidate items for inclusion in PREM-ED 65+. Subsequently, shortlisting and prioritisation of candidate items for inclusion in the final instrument is planned using a nominal groups technique. This will involve a range of stakeholders, including older adults and their carers [53]. The fourth and final step of development will then consist of psychometric field testing of the draft instrument. The anticipated result will be an instrument which meaningfully and usefully measures patient experience for older adults attending the ED.

## 5. Conclusions

Older adults are a significant and growing ED user group, both within the UK and internationally. Understanding their experiences is essential to ensuring the design and provision of ED services to meet their specific needs. This study utilised ‘insitu’ interviews carefully conducted immediately following emergency care to gain real time insights into patient experiences and needs.

Findings from this study confirm that older adults’ experiences of ED care can be categorised using a pre-existing ‘needs-based’ conceptual framework, although several new subthemes and an analytic theme emerged, which were not previously identified within a systematic review. Aside from providing discrete insight into the lived experience of older adults attending an ED, data from this study will inform a comprehensive list of items for inclusion, in a patient reported experience measure, named PREM-ED 65+.

## Figures and Tables

**Table 1:** Stepwise mixed methods approach to PREM ED 65+ development (note the current study is within step 2).

Step 1: Conceptualising Patient Experience in the ED	Systematic Review ○ Qualitative meta-synthesis ○ Derivation of conceptual framework of patient experience of ED care [25]
Step 2: Understanding experiences specific to older adults in the ED	Qualitative study ○ Part I: Interviews with patients aged over 65 years ○ Part II: Focus groups with ED staff (professional caregivers)
Step 3: Generation and Prioritisation of Candidate Items	Consensus Setting (Nominal Groups Technique) ○ Generation of initial candidate items from existing data (Steps 1 and 2) ○ Generation and prioritisation of candidate items
Step 4: Psychometric Field Testing	Administration of draft PREM to patients ○ Confirmation of structural validity and reliability

**Table 2:** Purposive Sampling Categories.

Gender	Male Female
Age	65–84 years (old age) 85+ years (very old age)
Presentation Type	Primary medical complaint Primary traumatic injury
Acuity	Australasian Triage Category ○ Triage Category 1–3 (Higher Acuity) ○ Triage Category 4–5 (Lower Acuity)
Frailty	Clinical Frailty Scale (CFS) ○ CFS 1–3 (Lower Frailty) ○ CFS 4–6 (Moderate Frailty) ○ CFS 7–9 (Severe Frailty)

**Table 3:** Interview Guide.

1. What do you feel has affected your experience of visiting the A&E Department today? a. likes/dislikes, areas for improvement, communication, emotional needs, technical competence of staff, waiting experience? 2. What did you expect from your A&E visit today? a. Understanding, Reassurance, Medication, Other symptomatic relief, onward care/referral to services? 3. How safe have you felt during your time in A&E today? a. Feelings of security and vulnerability, experience of mistakes/mishaps, medication safety, ability to speak up?

**Table 4:** Summary of participant characteristics.

	N (%)
Gender	
Female	15 (62.5)
Male	9 (37.5)
Age	
65–74 years	12 (50)
75–84 years	10 (41.7)
84 years and above	2 (8.3)
Highest level of Education	
Primary	10 (41.6)
Secondary/Vocational	7 (29.2)
Post-secondary (e.g., degree)	5 (20.8)
Acuity (Australian Triage Scale)	
2–3 (Very Urgent/Urgent)	9 (62.5)
4 (Lower Acuity)	15 (37.5)
Presentation Type	
Medical Illness	16 (66.6)
Traumatic Injury	8 (44.4)
Frailty (Clinical Frailty Scale)	
1–3 (Lower Frailty)	15 (62.5)
4–6 (Moderate Frailty)	9 (37.5)
7–9 (Higher Frailty)	0

**Table 5:** Analytical Themes and Sub-Themes.

Analytical Theme	Existing Sub-Theme	New Sub-Theme
Communication Needs	Interpersonal Communication Informational Communication	Social Communication
Emotional Needs	Acknowledging Uncertainty Recognising Suffering Providing Empowerment	Reassurance
Care Needs	Symptom Relief Procedural Care	
Waiting Needs	Impact of Crowding Comfort ^1^ (associated with waiting)	Waiting experience
Physical/Environmental Needs	Comfort ^1^ (associated with physical needs)	
Attitudes and Values of the Team (new)	-	Perceptions of teamwork Staff attitudes and professionalism

^1^ For the purposes of the conceptual model, ‘comfort’ is considered a single concept; however, comfort associated with waiting and comfort associated with physical needs are considered under the respective analytical theme.

## Data Availability

The data presented in this study are available on request from the corresponding author.

## Supplementary Materials

The following supporting information can be downloaded at: https://www.mdpi.com/article/10.3390/healthcare11050717/s1, Table S1: Table of Individual Study Participant Characteristics.
