# Supplementary material for: Initial Development of a Patient Reported Experience Measure for Older Adults Attending the Emergency Department: Part I—Interviews with Service Users
Source: Healthcare (Basel). 2023 Feb 28;11(5):717. doi: 10.3390/healthcare11050717 (PMC10000837; doi:10.3390/healthcare11050717)
Supplement: Supplementary file 1 [file healthcare-11-00717-s001.zip › healthcare-2196814-supplementary.pdf]

Table S1: Individual Study Participant Characteristics

Participant (P)	Gender	Age	ATS Category	CFS Category	Presentation	Residence	Recent Occupation	AP	Education	Long term conditions	Duration mm:ss
**1**	Female	67y10m	2	1	Headache	Own Home	Accounts Assistant	Yes	Nil formal	No	11:30
**2**	Female	70y9m	2	1	Chest Pain	Own Home	Nurse	Yes	Degree	Yes	13:03
**3**	Female	80y6m	3	4	Chest Pain	Own Home	Housewife	No	Nil formal	Yes	29:03
**4**	Female	70y9m	4	6	Hip Injury	Own Home	Housewife	No	Nil formal	Yes	17:11
**5**	Male	76y7m	3	4	Abdominal Pain	Own Home	Quarryman	No	Nil formal	Yes	22:53
**6**	Female	77y10m	3	6	Fall	Own Home	Housewife	No	Nil formal	Yes	19:30
**7**	Male	76y1m	3	1	Surgical Complication	Own Home	Administrator	No	Vocational	Yes	16:54
**8**	Male	83y11m	3	2	Epistaxis	Own Home	Builder	Yes (Partner)	Vocational	No	28:41
**9**	Female	70y7m	3	1	Head Injury	Own Home	Carer	Yes Daughter	Vocational	Yes	29:18
**11**	Female	82y9m	2	4	Chest Pain	Own Home	Archaeologist	No	Degree	Yes	29:03
**12**	Female	66y7m	3	1	Head Injury	Own Home	District Nurse	Yes (Husband)	Degree	Yes	41:47
**13**	Male	82y9m	2	5	Shortness of breath	Own Home	Civil Servant	No	Secondary Education	Yes	28:11

**14**	Male	85y6m	2	3	Palpitations	Own Home	Railway Worker	Yes (Daughter)	Vocational	Yes	35:25
**15**	Female	80y8m	3	3	Abdominal Pain	Own Home	Cashier	Yes (Spouse)	Nil formal	Yes	21:15
**16**	Female	83y	4	2	Fracture	Own Home	Shop Asst	Yes (Spouse)	Nil formal	Yes	27:03
**17**	Male	66y8m	2	1	Major Trauma	Own Home	Teacher	Yes (Spouse)	Degree	Yes	51:07
**18**	Female	68y1m	3	1	Syncope	Own Home	Cardiographer	Yes (Spouse)	Vocational	Yes	9:29
**19**	Male	89y1m	3	6	Fall	Annexe	Managing Director	Yes (Daughter)	Nil formal	Yes	24:29
**20**	Male	69y5m	2	6	Suspected sepsis	Own Home	Electrician	Yes (Spouse)	Vocational	Yes	28:64
**21**	Female	84y9m	2	1	Chest Pain	Own Home	Ret'd Housewife	Yes (Child)	Nil formal	Yes	18:15
**22**	Female	65y3m	3	2	Overdose (Accidental)	Own Home	Religious Minister	Yes (Spouse)	Degree	Yes	55:44
**23**	Male	66y	3	1	LRTI	Own Home	Financial Advisor	Yes (Spouse)	Degree	Yes	17:16
**24**	Female	66y4m	4	4	Ankle Injury	Own Home (Respite)	Retired	No	Vocational	Yes	25:03

**25**	Female	67y7m	2	3	Anxiety	Own Home	Housewife	Yes (Spouse)	Nil	Yes	24:25

Notes: ATS= Australasian Triage Score; CFS= Clinical Frailty Scale; AP= Accompanying Persons

Participant 10 chose to withdraw post- recruitment.
